# Proliferation inhibition and apoptosis induction of imatinib-resistant chronic myeloid leukemia cells via *PPP2R5C* down-regulation

**DOI:** 10.1186/1756-8722-6-64

**Published:** 2013-09-03

**Authors:** Qi Shen, Sichu Liu, Yu Chen, Lijian Yang, Shaohua Chen, Xiuli Wu, Bo Li, Yuhong Lu, Kanger Zhu, Yangqiu Li

**Affiliations:** 1 Institute of Hematology, Jinan University, Guangzhou 510632, China; 2 Key Laboratory for Regenerative Medicine of Ministry of Education, Jinan University, Guangzhou 510632, China; 3 Department of Hematology, the First Affiliated Hospital of Jinan University, Guangzhou 510632, China

**Keywords:** CML, Imatinib resistant, *PPP2R5C*, RNA interference, Apoptosis, Cell proliferation

## Abstract

Despite the success of imatinib and other tyrosine kinase inhibitors (TKIs), chronic myeloid leukemia (CML) remains largely incurable, and a number of CML patients die due to *Abl* mutation-related drug resistance and blast crisis. The aim of this study was to evaluate proliferation inhibition and apoptosis induction by down-regulating *PPP2R5C* gene expression in the imatinib-sensitive and imatinib-resistant CML cell lines K562, K562R (imatinib resistant without an *Abl* gene mutation), 32D-Bcr-Abl WT (imatinib-sensitive murine CML cell line with a wild type *Abl* gene) and 32D-Bcr-Abl T315I (imatinib resistant with a T315I *Abl* gene mutation) and primary cells from CML patients by RNA interference. *PPP2R5C* siRNAs numbered 799 and 991 were obtained by chemosynthesis. Non-silencing siRNA scrambled control (SC)-treated, mock-transfected, and untreated cells were used as controls. The *PPP2R5C* mRNA and protein expression levels in treated CML cells were analyzed by quantitative real-time PCR and Western blotting, and in vitro cell proliferation was assayed with the cell counting kit-8 method. The morphology and percentage of apoptosis were revealed by Hoechst 33258 staining and flow cytometry (FCM). The results demonstrated that both siRNAs had the best silencing results after nucleofection in all four cell lines and primary cells. A reduction in *PPP2R5C* mRNA and protein levels was observed in the treated cells. The proliferation rate of the *PPP2R5C*-siRNA-treated CML cell lines was significantly decreased at 72 h, and apoptosis was significantly increased. Significantly higher proliferation inhibition and apoptosis induction were found in K562R cells treated with *PPP2R5C*-siRNA799 than K562 cells. In conclusion, the suppression of *PPP2R5C* by RNA interference could inhibit proliferation and effectively induce apoptosis in CML cells that were either imatinib sensitive or resistant. Down-regulating *PPP2R5C* gene expression might be considered as a new therapeutic target strategy for CML, particularly for imatinib-resistant CML.

## Introduction

Chronic myeloid leukemia (CML) is a hematopoietic stem cell disorder that occurs because of t(9;22)(q34;q11) translocations. CML prognoses markedly improved after the introduction of Abl tyrosine kinase inhibitors (TKIs). Since its approval in 2001 for frontline CML management, imatinib has proven to be effective in achieving high remission rates and improving prognosis. However, up to 33% of patients will not achieve an optimal response. Most patients with CML treated with imatinib will relapse if treatment is withdrawn, and numerous CML patients die due to *Abl* mutation-related drug resistance and blast crisis. These circumstances have led researchers to develop a new generation of TKIs. Although second-generation TKIs, such as AMN107, appear to improve the treatment of CML, TKI resistance and relapse also frequently occur in patients. *de novo* and secondary TKI resistance are significant problems for CML [1-5]. Therefore, how to treat patients with CML who are resistant to Bcr-Abl tyrosine kinase inhibitors is an important and urgent issue for clinical hematology.

Moreover, TKIs have significant off-target inhibitory effects on multiple kinases. TKIs, through the off-target PPP2R5Cinhibition of kinases important for B-cell signaling, reduce memory B-cell frequency and induce significant impairment of B-cell responses in CML [6]. TKIs also impair T cell function e.g., imatinib impairs CD8+ T cells specifically directed against leukemia-associated antigen function [7].

Further advances in the treatment of CML may require the development of novel agents such as siRNAs that target specific CMLs or specific immunotherapies without significant toxicity that may have cooperative effects with TKIs [8,9]. siRNAs targeting the *Bcr-Abl* and multidrug-resistance (*MDR-1*) genes were used in an anti-CML study and demonstrated that a breakpoint-specific short-interfering RNA (siRNA) was capable of decreasing Bcr-Abl protein expression and antagonizing Bcr-Abl-induced biochemical activities [10-12].

Synthetic small interfering RNAs (siRNAs) are promising gene-targeting agents that have shown great potential, particularly for development as specific anti-leukemia treatment [13,14]. A combination of *c-raf* and *bcl-2* siRNAs induced apoptosis in HL-60, U937, and THP cell lines and increased chemosensitivity to etoposide and daunorubicin [15].

Recently, we were the first to show that a higher *PPP2R5C* expression level is found in peripheral blood mononuclear cells from chronic phase CML patients, and *PPP2R5C* expression is significantly decreased in patients who achieved CR [16]. *PPP2R5C* is a regulatory B subunit of protein phosphatase 2A (PP2A), which is one of the main serine-threonine phosphatases in mammalian cells, and it maintains cell homeostasis by counteracting most of the kinase-driven intracellular signaling pathways [17]. The *PPP2R5C* gene encodes five different spliced variants including B56γ1, B56γ2, B56γ3, B56γ5, B56γ6, and B56γ4, which is only found in mice. The locus for the functional *PPP2R5C* gene is at 14q32.2, and a nonfunctional B56γ1 pseudogene for *PPP2R5C* is located at 3p21.3 [16-18]. *PPP2R5C* plays a crucial role in cell proliferation, differentiation, and transformation based on its induction of the dephosphorylation of p53 at various residues [19]. It has been reported that the dynamic nuclear distribution of the B56γ3 regulatory subunit controls nuclear PP2A activity and may be responsible for the tumor-suppressive function of PP2A [18]. Recently, alterations in the *PPP2R5C* expression pattern that are associated with malignant transformation have been characterized in lung cancer, and the *PPP2R5C* mutation F395C disrupts the B56γ–p53 interaction [20].

To confirm the role of *PPP2R5C* in the proliferation of CML, we analyzed the effect of down-regulating *PPP2R5C* gene expression in imatinib-sensitive and imatinib-resistant chronic myeloid leukemia (CML) cell lines and primary cells from CML patients by RNA interference and confirmed the proliferation inhibition and apoptosis induction of PPP2R5C in CML cells.

## Methods

### Cell culture

Imatinib-sensitive K562 cells (Institutes for Biological Sciences Cell Resource Center, Chinese Academy of Sciences, Shanghai, China) carrying 210 kDa wild-type Bcr-Abl were grown in Roswell Park Memorial Institute (RPMI) 1640 medium (Gibco-BRL, Grand Island, NY, USA) with 10% fetal calf serum (FCS) (Sijiqing Co., Hangzhou, China) and maintained in a humidified incubator at 37°C and 5% CO_2_. Imatinib-resistant K562R cells (provided by Prof. Jingxuan Pan, Department of Pathophysiology, Zhongshan School of Medicine, Sun Yat-sen University, Guangzhou, China) carrying 210 kDa wild-type Bcr-Abl were routinely maintained in the same medium including 1 μM imatinib. 32D-Bcr-Abl WT, an imatinib-sensitive murine CML cell line carrying a wild type *Abl* gene, and 32D-Bcr-Abl T315I, an imatinib-resistant CML cell line carrying a T315I mutation in Bcr-Abl (provided by Prof. Lin Qiu, Harbin Institute of Hematolgy & Oncology, Harbin, China), were established and maintained in RPMI 1640 medium with 10% FCS as previously described [21]. In addition, PBMCs from two patients with newly diagnosed, untreated chronic phase CML (case 1: female, 18 years old, PB white blood cell number (WBC): 108.6 × 10^9^/L, PB blast + promyelocyts 10%, case 2: female, 30 years old, WBC: 208.53 × 10^9^/L, PB blast + promyelocytes 3%), which were obtained with consent (the procedures were conducted according to the guidelines of the Medical Ethics committee of the health bureau of Guangdong Province of China), were grown in RPMI 1640 with 15% FCS. All experiments were performed using cells in the exponential growth phase.

### siRNA design and synthesis

The siRNAs *PPP2R5C*-siRNA799 (Chinese patent number: ZL 201110340411.1) and *PPP2R5C*-siRNA991 (Chinese patent number: ZL 201110337837.1), which target domains in the sixth and between the eighth and ninth exons in the *PPP2R5C* gene (ACCESSION NM_178587), respectively, and a non-silencing siRNA scrambled control (SC) were designed with online software (http://www.invitrogen.com) and synthesized by Invitrogen (Carlsbad, CA, USA) [22]. An Alexa Red Oligo (Invitrogen) was used to measure transfection efficiency.

### Nucleofection

Cells were collected by centrifugation and resuspended at 2.5 × 10^6^ cells/100 μl for the CML cell lines and primary CML cells in the appropriate Nucleofector™ kit V solution (Amaxa Biosystems, Cologne, Germany) [23-26]. Malignant CML cells were nucleofected with 3 μg of the *PPP2R5C* siRNAs or a non-silencing scrambled control (SC) siRNA using the T-003 program of the Nucleofection Device II (Amaxa Biosystems). Mock-transfected cells nucleofected without siRNA were used as a negative control. After nucleofection, the cells were immediately mixed with 500 μl of pre-warmed culture medium and transferred into culture plates. The treated cells were incubated at 37°C for 3 days for cell proliferation, apoptosis and microarray analyses. Three independent experiments for the cell lines were performed every 24 h.

### RNA isolation, reverse transcription, real-time qRT–PCR

Total RNA was isolated from different samples (CML cell lines and primary CML cells) using TRIzol (Invitrogen). cDNA for qRT-PCR was synthesized using the Superscript II RNaseH Reverse Transcriptase Kit (Invitrogen). The expression level of *PPP2R5C* and the *β2-MG* reference gene was determined by SYBR Green I real-time PCR. PCR was performed as previously described [16]. The sequences of the primers used in qRT-PCR are as follows: *PPP2R5C*: 5′-GTAATAAAGCGGGCAGCAGG-3′ (forward) and 5′-CAAAGTCAAAGAGGACGCAACA-3′ (reverse) and *β*_*2*_*M*: 5′-CAGCAAGGAC TGGTCTTTCTAT-3′ (forward) and 5′-GCGGCATCTTCAAACCTC-3′ (reverse) [22].

### Immunoblotting

A total of 2 × 10^6^ K562 and K562R cells were collected 72 h after nucleofection, and proteins were extracted using a RIPA total protein lysate kit (Shennengbocai, Shanghai, China). Protein quantification was performed according to conventional methods. Protein samples (30 μg) were added to SDS loading buffer, heated at 100°C for 5 min, and then electrophoresed in 10% SDS-polyacrylamide gels at 100 V for 30 min followed by 120 V for 50 min (Bio-Rad). The separated proteins were transferred onto nitrocellulose membranes (Invitrogen) using a tank system (Bio-Rad). The membranes were blocked with 3% blocking reagent for 2 h and then incubated with polyclonal rabbit anti-human PPP2R5C antibody (1:200; Sigma, USA) or mouse anti-actin antibody (1:1000; Lianke, Hangzhou, China) followed by incubation with goat anti-rabbit or donkey anti-mouse IgG antibodies, respectively (Jackson ImmunoResarch, USA; Lianke, Hangzhou, China). Immunoreactive proteins were visualized by chemiluminescence (Lianke, Hangzhou, China), and images were obtained with a Vilber Lourmat system (UVI, UK). The expression level of PPP2R5C was calculated with image quantitation analysis software using *β-actin* as a reference gene [25,26].

### Cell proliferation assays

The proliferation of CML cell lines and primary CML cells was indirectly assayed using the CCK-8 kit (Dojindo, Japan), which stains living cells. After transfection, approximately 5 × 10^4^ cells in 100 μL, including control cells, were incubated in triplicate in 96-well plates. At 24, 48, and 72 h, the CCK-8 reagent (10 μL) was added to each well, and the cells were incubated at 37°C for 6 h. The optical density at 450 nm was measured using an automatic microplate reader (Synergy4; Bio-Tek, Winooski, VT, USA) [23].

### Apoptosis analysis

At 72 h post-transfection, 5 × 10^4^ of the CML cell lines and primary CML cells were fixed, washed twice with PBS, and stained with Hoechst 33258 staining solution according to the manufacturer’s instructions (Beyotime, Haimen, China). Changes in the nuclei of cells after Hoechst 33258 staining were observed with a confocal laser-scanning microscope (LSM 510 META DuoScan; Carl Zeiss, Germany). The cell lines (5 × 10^5^) were collected 48 and 72 h after transfection and then prepared with FITC-labeled anti-Annexin-V (BD Pharmingen, San Diego, CA, USA) and propidium iodide (Kaiji, Nanjing, China) according to the manufacturers’ protocol and measured by flow cytometry (Beckman Coulter, Fullerton, CA, USA). The results were analyzed using Windows MDI 2.9 software [23].

### Statistical analysis

Statistical analyses were performed with paired *t*-tests and one-way ANOVA using SPSS 11.5 statistical software. Kruskal-Wallis analysis was used to analyze the *PPP2R5C* mRNA levels in different samples. Differences were considered statistically significant at *p* < 0.05.

## Results

### *PPP2R5C*-specific siRNAs suppress *PPP2R5C* expression in CML cells

**Figure 1:**
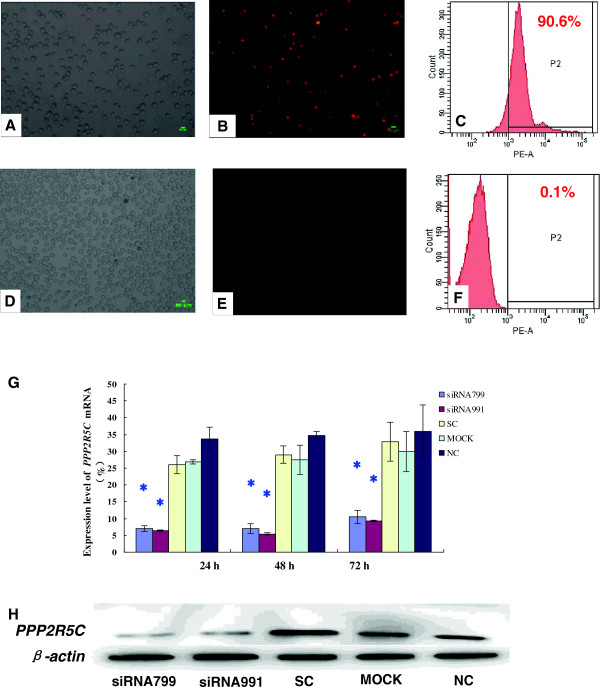
**Inhibition of *PPP2R5C *expression in K562 cells by RNA interference.** Detection of the transfection efficiencies in K562 cells by fluorescence microscopy (Bar = 50 μm) and flow cytometry (Positive cells are shown as the P2 domain). **A** and **B**: Alexa Red Oligo-transfected K562 cells (fluorescence microscope); **C**: Alexa Red Oligo-transfected K562 cells (FCM); **D** and **E**: Mock-transfected K562 cells (fluorescence microscope); **F**: Mock-transfected K562 cells (FCM). **G**: Suppression of *PPP2R5C* mRNA expression as measured by qRT–PCR after nucleofection with *PPP2R5C* siRNAs (3 μg). *, *p* < 0.05 compared with expression in cells treated with non-silencing control RNA. **H**: PPP2R5C protein level in K562 cells 72 h after nucleofection with *PPP2R5C* siRNAs (3 μg). Non-treated cells (nc), mock-transfected (mock), and scrambled control non-silencing RNA (SC)-treated cells were used as controls.

**Figure 2:**
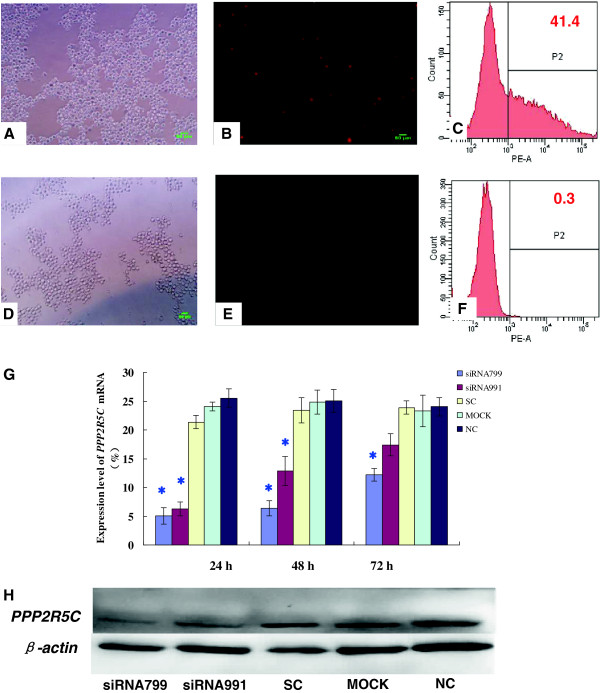
**Inhibition of *PPP2R5C *expression in K562R cells by RNA interference.** Detection of the transfection efficiencies in K562R cells by fluorescence microscopy (Bar = 50 μm) and flow cytometry (positive cells are shown in the P2 domain). **A** and **B**: Alexa Red Oligo-transfected K562R cells (fluorescence microscope); **C**: Alexa Red Oligo-transfected K562R cells (FCM); **D** and **E**: Mock-transfected K562R cells (fluorescence microscope); **F**: Mock-transfected K562R cells (FCM). **G**: Suppression of *PPP2R5C* mRNA expression as measured by qRT–PCR after nucleofection with *PPP2R5C* siRNAs (3 μg). *, *p* < 0.05 compared with expression in cells treated with control non-silencing RNA. **H**: PPP2R5C protein level in K562R cells 72 h after nucleofection with *PPP2R5C* siRNAs (3 μg). Non-treated cells (nc), mock-transfected (mock), and scrambled non-silencing control RNA (SC)-treated cells were used as controls.

We first verified transfection efficiency with Alexa Red Oligo-transfected K562, K562R, 32D-Bcr-Abl WT and 32D-Bcr-Abl T315I cell lines, which was 86.38 ± 6.82% (Figure 1), 40.52 ± 4.48% (Figure 2), 50.97 ± 4.36% (Additional file 1: Figure S1) and 63.26 ± 3.75% (Additional file 2: Figure S2), respectively, and Alexa Red Oligo-transfected primary CML cells, which was only 15.6%.

To determine the suppression of *PPP2R5C* expression in CML cells after siRNA treatment, *PPP2R5C* mRNA expression was analyzed by qRT–PCR 24, 48, and 72 h after nucleofection, while the suppression of PPP2R5C protein expression in K562 and K562R cells was analyzed by immunoblotting 72 h after nucleofection. *PPP2R5C*-siRNA799 and *PPP2R5C*-siRNA991 were measured from 24 to 72 h post-transfection. The *PPP2R5C* mRNA level in K562 cells was 7.00 ± 0.83 and 6.44 ± 0.87% at 24 h with *PPP2R5C*-siRNA799 and *PPP2R5C*-siRNA991 transfection, respectively, while the SC level was 26.11 ± 2.69% (Figure1)*.* The *PPP2R5C* mRNA level in K562R cells was 5.06 ± 1.47 and 6.23 ± 1.19% at 24 h with *PPP2R5C*-siRNA799 and *PPP2R5C*-siRNA991 transfection, respectively, while the SC level was 21.37 ± 1.17% (Figure 2)*.* Similar inhibition results were found for the protein levels. The PPP2R5C protein expression level was reduced by 55.26 and 52.58% in *PPP2R5C*-siRNA799- and *PPP2R5C*-siRNA991-treated K562 cells, respectively, compared with the control level at 72 h (Figure 1), while the reduction in treated K562R cells was 53.81 and 50.21%, respectively (Figure 2).

We also analyzed the suppression effect of both siRNAs in the murine CML cell lines 32D-Bcr-Abl WT and 32D-Bcr-Abl T315I. The *PPP2R5C* mRNA level in 32D-Bcr-Abl WT cells was 5.71 ± 2.45 and 8.88 ± 1.39% at 24 h with *PPP2R5C*-siRNA799 and *PPP2R5C*-siRNA991, respectively, while the SC level was 20.25 ± 1.37% (Additional file 1: Figure S1)*.* The *PPP2R5C* mRNA level in 32D-Bcr-Abl T315I cells was 3.14 ± 2.04 and 3.18 ± 1.13% at 24 h with *PPP2R5C*-siRNA799 and *PPP2R5C*-siRNA991, respectively, while the SC level was 12.04 ± 1.11% (Additional file 2: Figure S2)*.* A reduction in the *PPP2R5C* mRNA levels was also observed at 48-72 h. The *PPP2R5C* mRNA level in primary leukemic CML cells was decreased 4.71 and 6.09% at 24 h with *PPP2R5C*-siRNA799 and *PPP2R5C*-siRNA991, respectively, compared with the SC (14.01%)*.* A reduction in the *PPP2R5C* mRNA level was also observed at 48-72 h (Additional file 3: Figure S3). Although the 32D-Bcr-Abl WT and 32D-Bcr-Abl T315I cells are originally from a mouse, the *PPP2R5C* siRNAs target the same sequences in these cells due to homology of the mouse *PPP2R5C* and that of humans according to data from GenBank.

### *PPP2R5C* suppression inhibits proliferation and induces apoptosis in CML cells

**Figure 3:**
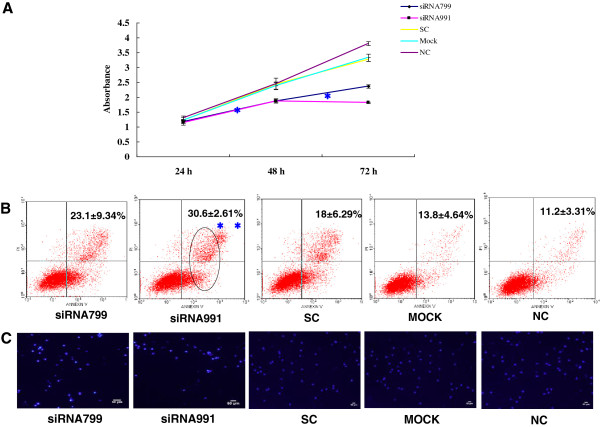
**Biological consequences of *PPP2R5C *silencing. (A)** Absorbance of *PPP2R5C*-siRNA799- and *PPP2R5C*-siRNA991-treated and control cells (K562) at different time points as measured by the CCK-8 method. The results represent mean values of three independent experiments. *, *p* < 0.001 compared with the scrambled non-silencing control RNA-treated cells. **(B)** Induction of apoptosis by *PPP2R5C* suppression in K562 cells 72 h after nucleofection with *PPP2R5C*-siRNA (3 μg). **, *p* = 0.004 compared with non-silencing control RNA-treated cells. **(C)** Hoechst 33258-stained K562 nuclei 72 h after transfection in the *PPP2R5C* siRNA groups were mostly densely stained, demonstrating a white color, while the normal nuclei in the control groups were light blue under a fluorescence microscope. Bar = 50 μm.

**Figure 4:**
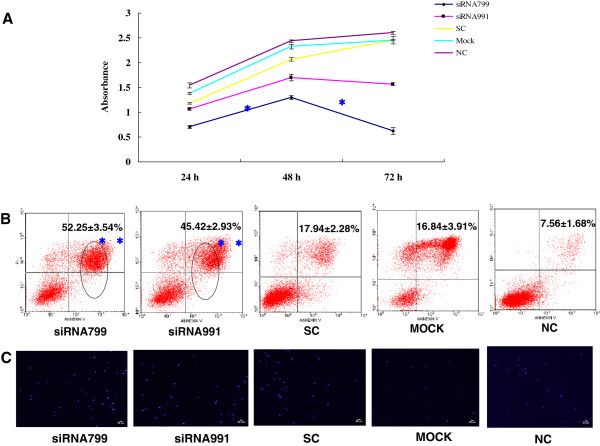
**Biological consequences of *PPP2R5C *silencing. (A)** Absorbance of *PPP2R5C*-siRNA799- and *PPP2R5C*-siRNA991-treated and control cells (K562R) at different time points as measured by the CCK-8 method. The results represent mean values of three independent experiments. *, *p* < 0.05 compared with scrambled non-silencing control RNA-treated cells. **(B)** Induction of apoptosis by *PPP2R5C* suppression in K562R cells 72 h after nucleofection with *PPP2R5C* siRNA (3 μg). **, *p* < 0.001 compared with non-silencing control RNA-treated cells. **(C)** Hoechst 33258-stained K562R nuclei 72 h after transfection in the *PPP2R5C* siRNA group were mostly densely stain, demonstrating a white color, while the normal nuclei in control groups were light blue under a fluorescence microscope. Bar = 50 μm.

**Figure 5:**
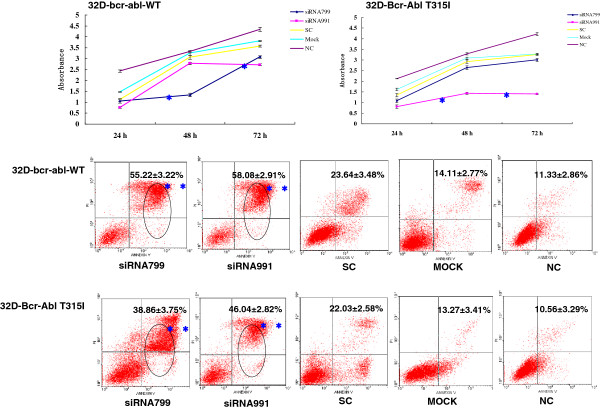
**Biological consequences of *PPP2R5C *silencing.** Absorbance of *PPP2R5C-*siRNA799- and *PPP2R5C*-siRNA991-treated and control cells (32D-bcr-abl-WT,32D-Bcr-Abl T315I) at different time points as measured by the CCK-8 method. The results represent mean values of three independent experiments. *, p < 0.001 compared with scrambled non-silencing control RNA-treated cells. Induction of apoptosis by *PPP2R5C* suppression in 32D-bcr-abl-WT and 32D-Bcr-Abl T315I cells 72 h after nucleofection with *PPP2R5C* siRNA (3 μg). *, *p* < 0.001,**, *p* = 0.005, ***, *p* = 0.001 compared with non-silencing control RNA-treated cells.

**Figure 6:**
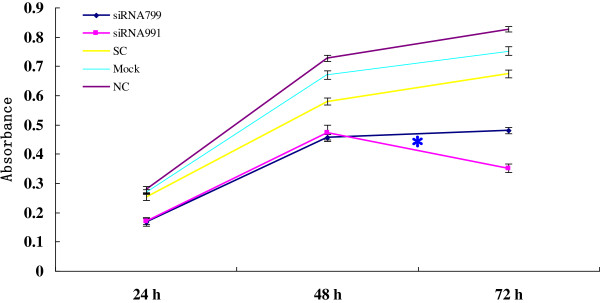
**Absorbance of *PPP2R5C*-siRNA799 and *PPP2R5C*-siRNA991-treated primary CML and control cells at different time points as measured by the CCK-8 method.** The results represent the mean values of three independent experiments. *, < 0.001 compared with non-silencing scrambled control RNA-treated cells.

The proliferation rate of K562 and K562R cells transfected with *PPP2R5C*-siRNA799 and *PPP2R5C*-siRNA991 was significantly decreased at 48-72 h compared with controls (*p* < 0.0001) (Figures 3 and 4). The proliferation rate of 32D-Bcr-Abl WT with *PPP2R5C*-siRNA799 was significantly decreased at 48-72 h, and it was significantly decreased at 72 h with *PPP2R5C*-siRNA991 (*p* < 0.0001) (Figure 5). The proliferation rate of the 32D-Bcr-Abl T315I cells with *PPP2R5C*-991-siRNA was significantly decreased at 48-72 h compared with controls (*p* < 0.0001) (Figure 5). The proliferation rate of primary CML cells with *PPP2R5C*-siRNA799 and *PPP2R5C*-siRNA991 was significantly decreased at 72 h (*p* < 0.0001) (Figure 6). K562 cells transfected with *PPP2R5C*-799-siRNA showed a significant increase in Annexin V/PI-positive cells (apoptosis) at 72 h, reaching 30.6 ± 2.61% (*p* = 0.04) (Figure 3). Moreover, the apoptotic (Annexin V/PI-positive cells) rate of K562R cells transfected with *PPP2R5C*-799-siRNA and *PPP2R5C*-991-siRNA showed a significant increase at 72 h, reaching 52.25 ± 3.54 and 45.42 ± 2.93%, respectively (*p* < 0.0001) (Figure 4). Similar results were found for 32D-Bcr-Abl WT and 32D-Bcr-Abl T315I cells transfected with *PPP2R5C*-siRNA799 and *PPP2R5C*-siRNA991. The apoptotic rate of 32D-Bcr-Abl WT cells was significantly increased at 72 h, reaching 55.25 ± 3.22 and 58.08 ± 2.91% with *PPP2R5C*-siRNA799 and *PPP2R5C*-siRNA991, respectively (*p* < 0.0001) (Figure 5), while it reached 38.86 ± 3.75 (*PPP2R5C*-siRNA799) and 46.04 ± 2.82% (*PPP2R5C*-siRNA991) in 32D-Bcr-Abl T315I cells treated for 72 h (*p* =0.005, *p* =0.001) (Figure 5). Furthermore, morphological changes consistent with apoptosis were observed by Hoechst staining (Figures 3 and 4).

**Figure 7:**
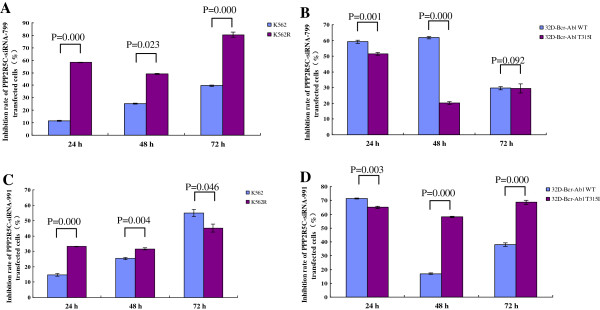
**The proliferation inhibition effect of *PPP2R5C*-siRNAs on the imatinib-sensitive and imatinib-resistant cell lines. A**: K562 and K562R cells treated with *PPP2R5C*-siRNA799, **B**: 32D-Bcr-Abl WT and 32D-Bcr-Abl T315I cells treated with *PPP2R5C*-siRNA799, **C**: K562 and K562R cells treated with *PPP2R5C*-siRNA991, **D**: 32D-Bcr-Abl WT and 32D-Bcr-Abl T315I cells treated with *PPP2R5C*-siRNA991.

**Figure 8:**
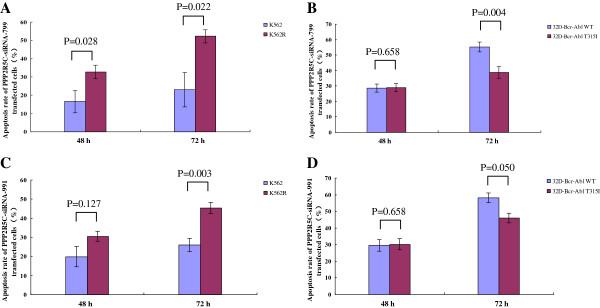
**The apoptosis induction effect of *PPP2R5C*-siRNAs on imatinib-sensitive and imatinib-resistant cell lines. A**: K562 and K562R cells treated with *PPP2R5C*-siRNA799, **B**: 32D-Bcr-Abl WT and 32D-Bcr-Abl T315I cells treated with *PPP2R5C*-siRNA799, **C**: K562 and K562R cells treated with *PPP2R5C*-siRNA991, **D**: 32D-Bcr-Abl WT and 32D-Bcr-Abl T315I cells treated with *PPP2R5C*-siRNA991.

We next compared the effect of the *PPP2R5C* siRNAs on imatinib-sensitive and imatinib-resistant cell lines, and interestingly, significantly higher proliferation inhibition and apoptosis induction were found for K562R cells treated with *PPP2R5C-*siRNA799 than for K562 cells (Figures 7 and 8). In contrast, the inhibition of proliferation and apoptosis was higher for 32D-Bcr-Abl WT than 32D-Bcr-Abl T315I cells treated with the same siRNA (Figures 7 and 8). However, the effect of *PPP2R5C*-siRNA991 on the inhibition of proliferation and apoptosis induction appeared variable between the imatinib sensitive and resistant cells (Figures 7 and 8).

## Discussion

Targeted therapies are directed at unique molecular signatures of cancer cells to produce greater efficacy with less toxicity. The development and use of such therapeutics allow us to practice personalized medicine and improve cancer care [27]. Imatinib is the first successful molecular drug specifically targeting the *Abl* gene and has proven to be effective in achieving high remission rates and improving CML prognosis. Because TKI resistance and relapse frequently occur in patients, new targeted drugs that can specifically inhibit TKI-resistant CML urgently need to be developed. RNAi represents a new alternative for CML treatment that overcomes the difficulties of current drug treatments such as acquired resistance. The therapeutic targeting of *Bcr-Abl* transcripts by siRNA was demonstrated in imatinib-resistant CML cells [10,28].

PPP2R5C plays a crucial role in cell proliferation, differentiation, and transformation based on its induction of the dephosphorylation of p53 at various residues [19] and may be responsible for the tumor-suppressive function of PP2A [18]. To confirm the role of PPP2R5C down-regulation on the inhibition of CML cells, particularly TKI-resistant CML cells, we used two *PPP2R5C* siRNAs that target different exon sequences to analyze their effect on the inhibition of proliferation and apoptosis induction in CML cells. Moreover, to investigate the *PPP2R5C* siRNA effects in imatinib-resistant CML cells, we selected two pairs of CML cell lines, including the imatinib-sensitive cell lines K562 and 32D-Bcr-Abl WT and the imatinib-resistant cell lines K565R, which lacks an *Abl* mutation, and 32D-Bcr-Abl T315I, which has an T315I *Abl* mutation, to compare the different changes induced by *PPP2R5C* siRNA.

In general, RNAi effects are detected between 24 and 72 h after siRNA transfection. We demonstrated that the siRNAs effectively silenced *PPP2R5C* post-transcriptionally, and the control siRNA had no obvious influence 72 h after nucleofection. These results were confirmed at the RNA and protein levels. siRNAs targeting different exon domains had different efficacies for *PPP2R5C* gene silencing and subsequent biological consequences. Both *PPP2R5C* siRNAs demonstrated significant effects on the knockdown of PPP2R5C expression in CML cell lines, and *PPP2R5C*-siRNA799, which targets exon six, demonstrated robust knockdown of *PPP2R5C* expression in K562R cells at all time points.

There are reports that siRNAs targeting *Bcr-abl* increased sensitivity to imatinib in Bcr-Abl-overexpressing cells and cells expressing the imatinib-resistant Bcr-Abl kinase domain mutations H396P and Y253F [10,28]. There are no reports regarding the effects of the suppression of *PPP2R5C* on changing cell biological functions. Our previous study first demonstrated that the suppression of *PPP2R5C* by RNAi effectively inhibited the proliferation of the Molt-4 and Jurkat cell lines; however, the suppression of *PPP2R5C* by RNAi could not significantly induce apoptosis in Molt-4 and Jurkat T cells [22]. In contrast, the *PPP2R5C* siRNAs not only inhibited cell proliferation but also induced apoptosis in imatinib-sensitive and imatinib-resistant CML cell lines. These results indicated that down-regulating PPP2R5C could significantly inhibit the proliferation of CML cells, and the underlying mechanism might be different between CML and T-ALL cells. More importantly, we found a significantly higher inhibition effect in K562R cells treated with *PPP2R5C*-siRNA799, and the inhibition effect in 32D-Bcr-Abl T315I cells, which have a T315I *Abl* mutation, was similar to that of 32D-Bcr-Abl WT cells. Such effects are particularly important for the targeted therapy of imatinib-resistant CML cells that either lack an *Abl* mutation and have primary and imatinib-induced resistance or those with an *Abl* T315I mutation, which resist new-generation TKIs. Therefore, it is interesting to analyze the molecular mechanism of *PPP2R5C* siRNA-mediated cell proliferation suppression in different leukemia cells. It has been reported that TKI-resistant, Philadelphia chromosome-positive cell lines without an *Abl* mutation are unique because they dephosphorylate ERK1/2 and STAT5 after imatinib treatment, while PI3K/AKT1/mTOR activity remains unaffected. The inhibition of AKT1 leads to apoptosis in imatinib-resistant cell lines. Therefore, these Ph + cell lines show a form of imatinib-resistance attributable to the constitutive activation of the PI3K/AKT1 pathway [3]. Whether down-regulating PPP2R5C contributes similar effects to the inhibition of PI3K/AKT1/mTOR signaling requires further investigation. We also found that *PPP2R5C* siRNA could inhibit the proliferation of primary CML cells in limited experiments, and this effect should be further explored using a larger patient cohort.

Moreover, whether *PPP2R5C* siRNAs could potentiate the efficacy of TKIs in imatinib-resistant cells is worthy of further investigation. One study has shown a synergistic effect between AMN107 and arsenic trioxide (ATO) or *Bcr-Abl*-siRNA in the K562R imatinib-resistant cells or those with an H396P abl mutation, indicating that the combination of AMN107 and ATO or siRNA may represent a new strategy for the treatment of imatinib-resistant CML patients [10,29].

In conclusion, our findings provide evidence for the effect of proliferation inhibition and apoptosis induction in CML cells by PPP2R5C knockdown, and such effects may particularly benefit developing a strategy including a combination of targeted therapy using TKIs for resistant cells. A successful clinical trial demonstrated that the in vivo application of targeted nonvirally delivered synthetic *Bcr-abl* siRNA in a female patient with recurrent CML that was imatinib resistant (Y253F mutation) and chemotherapy after an allogeneic hematopoietic stem cell transplantation could silence the expression of the *Bcr-Abl* gene [28]. These data imply that siRNA may be suitable for development as a specific anti-leukemia treatment. However, a recent study demonstrated that the phosphatase activity of PP2A is suppressed in chronic myeloid leukemia and other malignancies characterized by aberrant oncogenic kinase activity, and preclinical studies show that the pharmacological restoration of PP2A tumor-suppressor activity by PP2A-activating drugs (e.g., FTY720) effectively antagonizes cancer development and progression [30]. These findings appear to be contrary to our results, and further characterization of the function of the different regulatory B subunits of PP2A and a discussion of the different effects on the different PP2A target subunits is needed.

## Competing interests

The authors declare that they have no competing interests.

## Authors' contributions

YQL contributed to concept development and study design. QS performed the nucleofection, real-time PCR, cell proliferation assays and apoptosis analysis, SCL performed the immunoblotting and apoptosis analysis, YC screened the highly efficient and specific *PPP2R5C* siRNAs, and LJY, SHC, XLW and BL helped to collect samples and perform cell culture. YHL and KEZ was responsible for the collection of clinical data. YQL and QS coordinated the study and helped draft the manuscript. All authors read and approved the final manuscript.

## Supplementary Material

Additional file 1: Figure S1 Inhibition of *PPP2R5C* expression in 32D-bcr-abl-WT cells by RNA interference. Alexa Red Oligo-transfected (A) and mock-transfected (B) 32D-bcr-abl-WT cells (B) 11 h after transfection as measured with FCM (Positive cells are shown as the P2 domain). (C) Suppression of *PPP2R5C* mRNA expression as measured by qRT–PCR after nucleofection with *PPP2R5C* siRNAs (3 μg) compared with expression in cells treated with non-silencing control RNA. Click here for file

Additional file 2: Figure S2 Inhibition of *PPP2R5C* expression in 32D-Bcr-Abl T315I cells by RNA interference. Alexa Red Oligo-transfected (A) and mock-transfected (B) 32D-Bcr-Abl T315I cells 11 h after transfection as measured with FCM (positive cells are shown in the P2 domain). (C) Suppression of *PPP2R5C* mRNA expression as measured by qRT–PCR after nucleofection with *PPP2R5C* siRNAs (3 μg) compared with expression in cells treated with non-silencing control RNA. Click here for file

Additional file 3: Figure S3 Inhibition of *PPP2R5C* expression in primary CML cells by RNA interference. A: CML cells from a case with chronic phase CML treated with Alexa Red Oligo 11 h after transfection as measured by FCM (positive cells are shown in the P2 domain) with mock-transfected primary CML cells used as control (B). (C) Suppression of *PPP2R5C* mRNA expression as measured by qRT–PCR after nucleofection with *PPP2R5C* siRNAs (3 μg) compared with expression in cells treated with non-silencing control RNA. Click here for file
