# Supplementary figures and images for: Proliferation inhibition and apoptosis induction of imatinib-resistant chronic myeloid leukemia cells via PPP2R5C down-regulation
Source: J Hematol Oncol. 2013 Sep 3;6:64. doi: 10.1186/1756-8722-6-64 (PMC3847136; doi:10.1186/1756-8722-6-64)

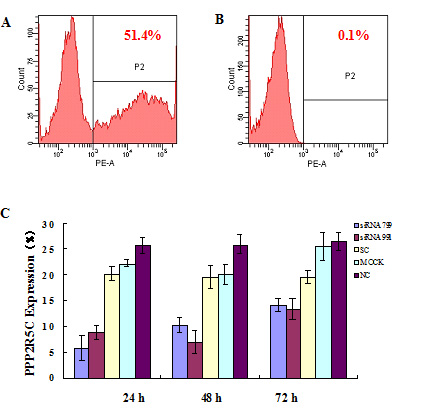

Supplement: Additional file 1: Figure S1 — Inhibition of PPP2R5C expression in 32D-bcr-abl-WT cells by RNA interference. Alexa Red Oligo-transfected (A) and mock-transfected (B) 32D-bcr-abl-WT cells (B) 11 h after transfection as measured with FCM (Positive cells are shown as the P2 domain). (C) Suppression of PPP2R5C mRNA expression as measured by qRT–PCR after nucleofection with PPP2R5C siRNAs (3 μg) compared with expression in cells treated with non-silencing control RNA. [file 1756-8722-6-64-S1.jpeg]

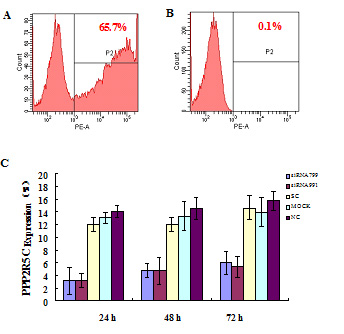

Supplement: Additional file 2: Figure S2 — Inhibition of PPP2R5C expression in 32D-Bcr-Abl T315I cells by RNA interference. Alexa Red Oligo-transfected (A) and mock-transfected (B) 32D-Bcr-Abl T315I cells 11 h after transfection as measured with FCM (positive cells are shown in the P2 domain). (C) Suppression of PPP2R5C mRNA expression as measured by qRT–PCR after nucleofection with PPP2R5C siRNAs (3 μg) compared with expression in cells treated with non-silencing control RNA. [file 1756-8722-6-64-S2.jpeg]

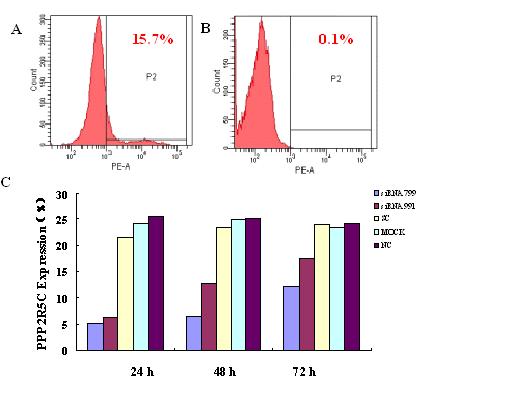

Supplement: Additional file 3: Figure S3 — Inhibition of PPP2R5C expression in primary CML cells by RNA interference. A: CML cells from a case with chronic phase CML treated with Alexa Red Oligo 11 h after transfection as measured by FCM (positive cells are shown in the P2 domain) with mock-transfected primary CML cells used as control (B). (C) Suppression of PPP2R5C mRNA expression as measured by qRT–PCR after nucleofection with PPP2R5C siRNAs (3 μg) compared with expression in cells treated with non-silencing control RNA. [file 1756-8722-6-64-S3.jpeg]
